# Supplementary material for: Evaluation of the S-locus in Prunus domestica, characterization, phylogeny and 3D modelling
Source: PLoS One. 2021 May 13;16(5):e0251305. doi: 10.1371/journal.pone.0251305 (PMC8118244; doi:10.1371/journal.pone.0251305)
Supplement: S1 Table — (DOCX) [file pone.0251305.s001.docx]

S1 Table. *S* alleles, specie name and accession number for each sequence obtained from NCBI used for the construction of the phylogenetic tree in Fig 4.

| **S allele** | **Species** | **NCBI code** |
| --- | --- | --- |
| S1 | *Prunus domestica* | MW407936.1 |
| S2 | *Prunus domestica* | EU113267.1 |
| S3 | *Prunus domestica* | MW407937.1 |
| S4 | *Prunus domestica* | MW407939.1 |
| S5 | *Prunus domestica* | MW407935.1 |
| S6 | *Prunus domestica* | AM746947.1 |
| S7 | *Prunus domestica* | AM992050.1 |
| S8 | *Prunus domestica* | CAQ51498.1 |
| S9 | *Prunus domestica* | AM746948.1 |
| S10 | *Prunus domestica* | MW407940.1 |
| S11 | *Prunus domestica* | MW407941.1 |
| S12 | *Prunus domestica* | MW407942.1 |
| S13 | *Prunus domestica* | MW407943.1 |
| S14 | *Prunus domestica* | MW407944.1 |
| S15 | *Prunus domestica* | MW407945.1 |
| S16 | *Prunus domestica* | MW407946.1 |
| S17 | *Prunus domestica* | MW407938.1 |
| S1 | *Prunus cerasifera* | AM992048.1 |
| S2 | *Prunus cerasifera* | AM992049.1 |
| S3 | *Prunus cerasifera* | AM746943.1 |
| S4 | *Prunus cerasifera* | AM992050.1 |
| S5 | *Prunus cerasifera* | AM992051.1 |
| S6 | *Prunus cerasifera* | AM992052.1 |
| S7 | *Prunus cerasifera* | AM992053.1 |
| S8 | *Prunus cerasifera* | AM992054.1 |
| S9 | *Prunus cerasifera* | AM746944.1 |
| S10 | *Prunus cerasifera* | AM746945.1 |
| S11 | *Prunus cerasifera* | AM992055.1 |
| S12 | *Prunus cerasifera* | AM992056.1 |
| S13 | *Prunus cerasifera* | AM992057.1 |
| S14 | *Prunus cerasifera* | AM992058.1 |
| Unknown_M4 | *Prunus cerasifera* | CAQ51498.1 |
| S1 | *Prunus spinosa* | EF636467.1 |
| S3 | *Prunus spinosa* | DQ677584.1 |
| S7 | *Prunus spinosa* | DQ677586.1 |
| S8 | *Prunus spinosa* | DQ677587.1 |
| S9 | *Prunus spinosa* | DQ677588.1 |
| S10 | *Prunus spinosa* | DQ677589.1 |
| S12 | *Prunus spinosa* | DQ677590.1 |
| S15 | *Prunus spinosa* | EF636468.1 |
| S16 | *Prunus spinosa* | EF636469.1 |
| S19 | *Prunus spinosa* | EF636470.1 |
| S24 | *Prunus spinosa* | EF636471.1 |
| S5 | *Prunus virginiana* | JQ627793.1 |
| S12 | *Prunus virginiana* | JQ627800.1 |
| S18 | *Prunus virginiana* | JQ627806.1 |
| S13 | *Prunus armeniaca* | EF062341.1 |
| S17 | *Prunus armeniaca* | EU516388.1 |
| S19 | *Prunus armeniaca* | EF133689.1 |
| S24 | *Prunus armeniaca* | HQ615602.1 |
| S40 | *Prunus armeniaca* | GU354239.1 |
| S52 | *Prunus armeniaca* | KF951503.2 |
| Sc | *Prunus armeniaca* | ABO34168.1 |
| S3 | *Prunus dulcis* | KY059853.1 |
| S10 | *Prunus dulcis* | AM231658.1 |
| S11 | *Prunus dulcis* | AM231660.1 |
| S15 | *Prunus dulcis* | AM231664.1 |
| S21 | *Prunus dulcis* | EF690369.1 |
| S23 | *Prunus dulcis* | FN429354.1 |
| Sf | *Prunus dulcis* | AB467371 |
| S9 | *Pyrus pyrifolia* | AB104909.1 |
| S10 | *Pyrus pyrifolia* | AY158069.1 |
| S15 | *Pyrus pyrifolia* | AY249430.2 |
| S18 | *Pyrus pyrifolia* | KY608870.1 |
| S34 | *Pyrus pyrifolia* | DQ269500.1 |
| S44 | *Pyrus pyrifolia* | KY608875.1 |
| S4sm | *Pyrus pyrifolia* | AB009385.1 |
| S1 | *Prunus avium* | AY851457.1 |
| S5 | *Prunus avium* | AJ298314.1 |
| S6' | *Prunus avium* | AAT72120.1 |
| S12 | *Prunus avium* | AY259115.1 |
| S18 | *Prunus avium* | DQ508941.1 |
| S21 | *Prunus avium* | DQ508944.1 |
| S38 | *Prunus avium* | JQ280516.1 |
| S1 | *Prunus salicina* | AF433649.1 |
| S3 | *Prunus salicina* | AF432417.1 |
| S4 | *Prunus salicina* | AF432418.1 |
| S5 | *Prunus salicina* | AF433647.1 |
| S6 | *Prunus salicina* | AF433648.1 |
| S7 | *Prunus salicina* | AY781290.1 |
| S8 | *Prunus salicina* | AY902455.1 |
| S9 | *Prunus salicina* | AY996051.1 |
| S10 | *Prunus salicina* | DQ003310.1 |
| S11 | *Prunus salicina* | DQ512908.1 |
| S12 | *Prunus salicina* | DQ512909.1 |
| S15 | *Prunus salicina* | EF177346.1 |
| S16 | *Prunus salicina* | EF177347.1 |
| S17 | *Prunus salicina* | EF177348.1 |
| S18 | *Prunus salicina* | EF177349.1 |
| S19 | *Prunus salicina* | EU113259.1 |
| S20 | *Prunus salicina* | EU113260.1 |
| S21 | *Prunus salicina* | EU113261.1 |
| S22 | *Prunus salicina* | EU113262.1 |
| S23 | *Prunus salicina* | EU113263.1 |
| S24 | *Prunus salicina* | EU113264.1 |
| S25 | *Prunus salicina* | EU113265.1 |
| S26 | *Prunus salicina* | EU113266.1 |
| S27 | *Prunus salicina* | EU113267.1 |
| S32 | *Prunus salicina* | GU574195.1 |
| Se | *Prunus salicina* | BAF91848.1 |
| S41 | *Prunus salicina* | GU968758.1 |
| S1 | *Prunus persica* | DQ269994.1 |
| S31 | *Prunus speciosa* | GU968645.1 |
