# Supplementary material for: Evaluation of the S-locus in Prunus domestica, characterization, phylogeny and 3D modelling
Source: PLoS One. 2021 May 13;16(5):e0251305. doi: 10.1371/journal.pone.0251305 (PMC8118244; doi:10.1371/journal.pone.0251305)
Supplement: S1 Raw images — (PDF) [file pone.0251305.s002.pdf]

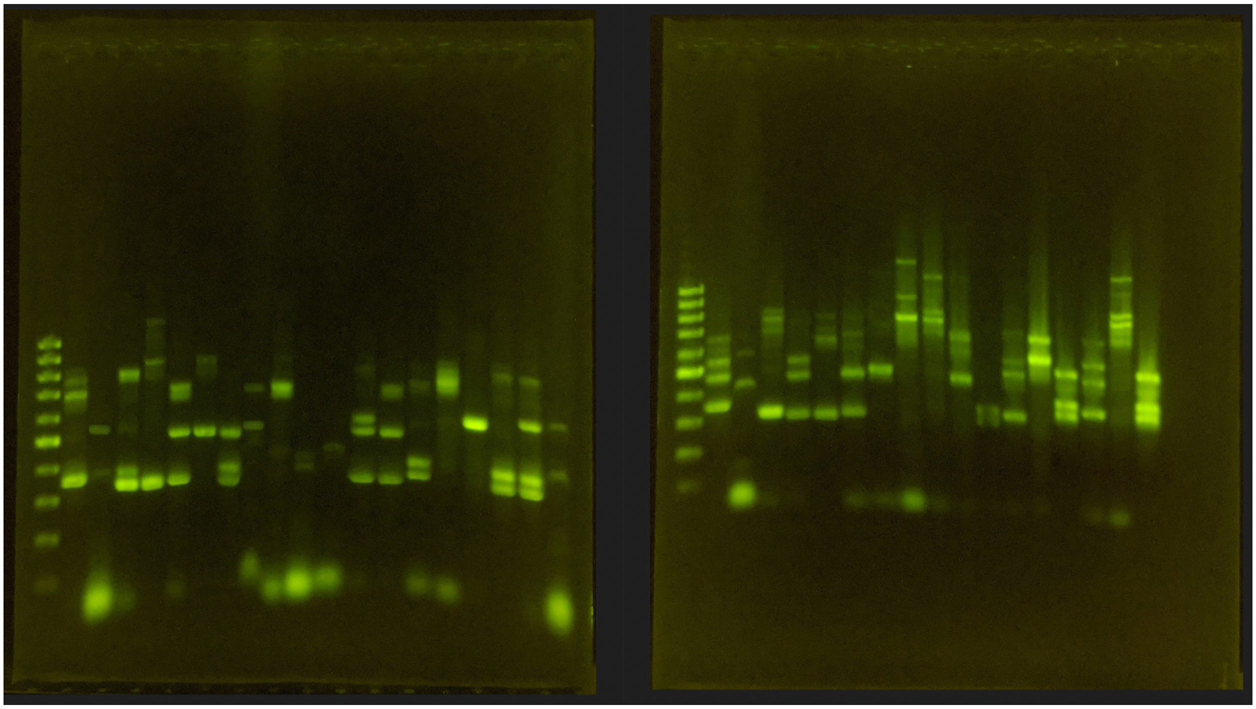

Original picture of the gel corresponding to Picture 2 in the manuscript.

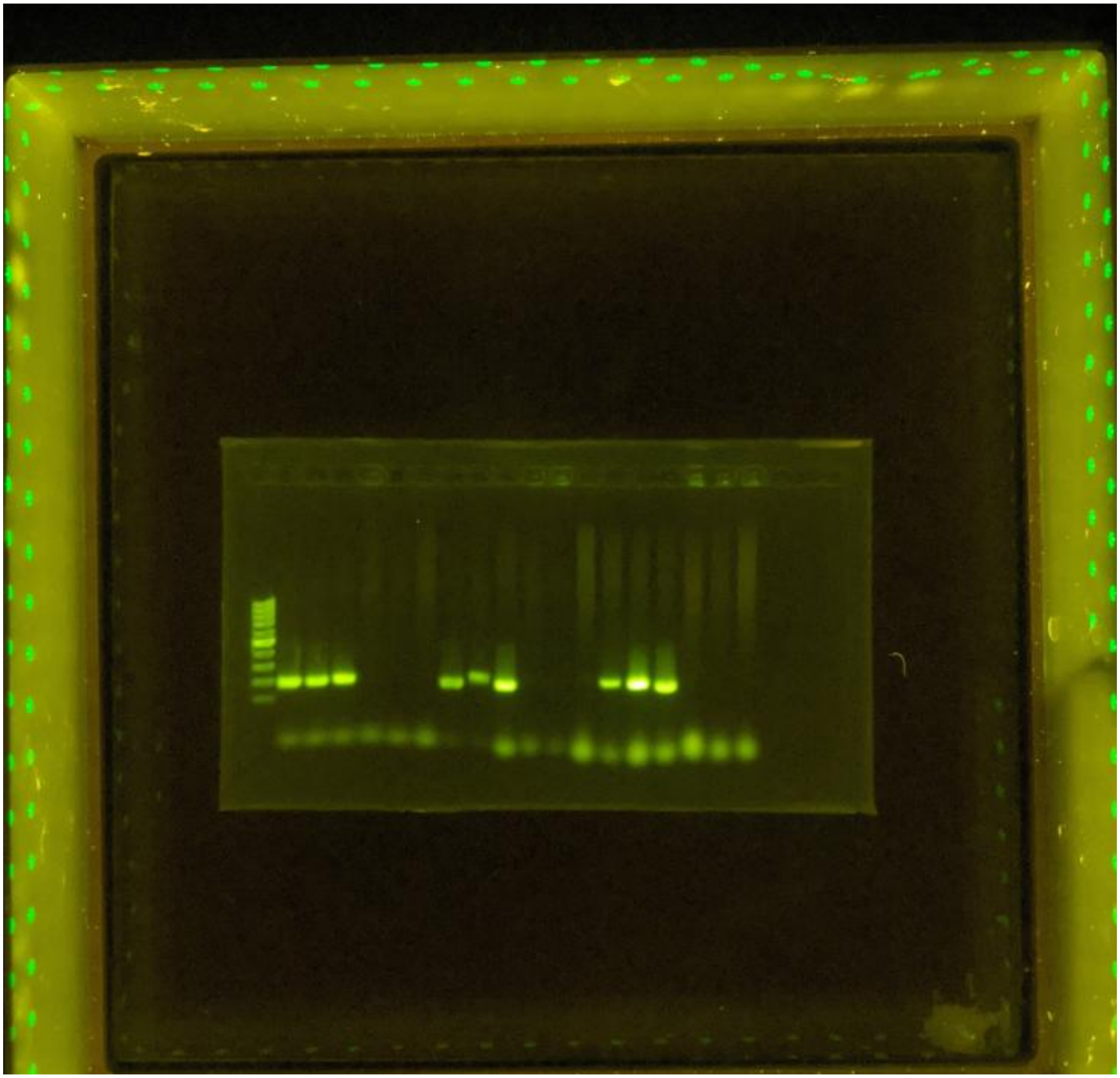

Original picture of the gel corresponding to Picture 3 in the manuscript.
From this image, we just want to show the efficiency of our new discovered molecular marker. We haven chosen to include in the manuscript the first part of the gel (lane 1 to 6), since in the middle, lane 8, we have included a positive control with another marker, and in lane 18, a negative control with water. Thus, the picture included in our manuscript corresponds to lanes 1 to 6 from this gel.
