# Supplementary material for: Evaluation of the S-locus in Prunus domestica, characterization, phylogeny and 3D modelling
Source: PLoS One. 2021 May 13;16(5):e0251305. doi: 10.1371/journal.pone.0251305 (PMC8118244; doi:10.1371/journal.pone.0251305)
Supplement: Attachment — Submitted filename: Reviewer.pdf [file pone.0251305.s003.pdf]

Reviewer

by Reviewer .

Submission date: 20-Mar-2021 11:46PM (UTC-0700)
Submission ID: 1538132062
File name: FiM_2021.docx (79.45K)
Word count: 9365
Character count: 50459

Evaluation of the *S*-locus in *Prunus domestica*, characterization, phylogeny and 3D modelling

Angel Fernandez i Marti[1*], Sarah Castro[2], Theodore M. DeJong[2] and Richard S. Dodd[1]

[1]Environmental Science, Policy and Management, University of California, Berkeley, United States
[2] Plant Science, University of California, Davis, United States

[*]Correspondence, afernandezmarti@berkeley.edu

Abstract,

Self-compatibility has become the primary objective of most prune (*Prunus domestica*) breeding programs in order to avoid the problems related to the gametophytic self-incompatibility (GSI) system present in this crop. GSI is typically under the control of a specific locus., known as the *S*-locus., which contains at least two genes. The first gene encodes glycoproteins with RNase activity in the pistils., and the second is an SFB gene expressed in the pollen. There is limited information on genetics of SI/SC in prune and in comparison., with other *Prunus* species, cloning., sequencing and discovery of different *S*-alleles is very scarce. Clear information about *S*-alleles can be used for molecular identification and characterization of the *S*-haplotypes. We determined the *S*-alleles of 36 cultivars and selections using primers that revealed 17 new alleles. In addition, our study describes for the first time the association and design of a molecular marker for self-compatibility in *P. domestica*. Our phylogenetic tree showed that the *S*-alleles are spread across the phylogeny, suggesting that like previous alleles detected in the Rosaceae., they were of trans-specific origin. We provide for the first time 3D models for the *P. domestica* SI RNase alleles as well as in other *Prunus* species, including *P. salicina* (Japanese plum), *P. avium* (cherry), *P. armeniaca* (apricot), *P. cerasifera* and *P. spinosa*.

Keywords, Plum, Self-(in)compatibility, *S*-genotype, Evolution, 3D modelling

Introduction

Self-incompatibility (SI) is an important biological feature of flowering plants, in which pollen-pistil interactions prevent self-fertility and determine the success of mating in out-crossed plants. The mechanism takes on special significance in agricultural fruit and nut crops, where SI reduces the potential for successful fertilization in minimally genetically diverse orchards. For such species, fruit and nut breeders commonly select for self-compatibility that has the added advantage of reduced dependence on insect pollinators.

Prunus, which belongs to the Rosaceae family, is one of the most important genera of fruit and nut trees that includes several commercially important species. Because successful fertilization is critical for fruit and nut production, the genus has been the subject of many studies of the genetic mechanism of SI and the identification of SI groups that can aid in orchard design. Fruit production is controlled by a gametophytic SI (GSI) system that is regulated by a single polymorphic locus containing at least two linked genes; one specifically expressed in the pistil and the other in the pollen (Kao and Tsukamoto, 2004). In diploid species, pollen tube growth is arrested in the style whenever the single *S* haplotype expressed in pollen matches one of the two *S* haplotypes expressed in the diploid pistil tissue (de Nettancourt 1977). The pistil component of SI in the Rosaceae, as in the Solanaceae and Plantaginaceae, is a ribonuclease, *S*-RNase (McClure et al. 1989; Tao et al. 1997), which acts as a cytotoxin in self-pollen tubes, whereas the candidate gene for the pollen component in all *Prunus* species was identified by Ushijima et al. (2003), to be a specific F-box (SFB) gene that is tightly linked to the S-RNase gene (Entani et al., 2003; Ikeda et al. 2005). However, to date, the role of SFB proteins is not completely understood. Extensive studies of self-(in)compatibility conducted on diploid *Prunus* species identified a T2-type RNase (Igic and Kohn 2001), with five conserved domains (C1, C2, C3, RC4 and C5) and one hyper-variable region (Sassa et al., 1996; Tao et al, 1999; Ushijima et al. 1998). Amino acid sequences of the different S-RNase alleles reveal a high degree of diversity within the same species.

As would be expected for proper functioning of the GSI system, co-evolution between pollen and stigma parts of the *S*-locus would require mutations in one part to be complemented by mutations in the other (Sutherland et al. 2008), leading to expectations of high polymorphism at both loci under frequency dependent selection. Indeed, high S-RNase allelic diversity has been detected for most species studied and high pollen part polymorphism has been reported, for example for *P. dulcis* (Ushijima et al. 2003), *P. avium* (Vaughan et al. 2006) and for several additional *Prunus* spp. (Sutherland et al. 2008). The pistil *S*-RNases are believed to be ancestral in most eudicots (Igic and Kohn 2006), however, diversification of the pollen *S* F-box proteins appears to be more taxon-dependent (Ushijima et al. 2003, Aguiar et al. 2015, Kubo et al. 2015, Akagi et al 2016), perhaps as a result of the rapid birth/death of F-box genes (Xu et al. 2009).

Breakdown of the GSI system results in self-compatible (SC) genotypes that are of great interest to the horticultural industry. The presence of haplotypes conferring SC has been studied in diploid *Prunus* species, including almond (*P. dulcis*), Japanese plum (*P. salicina*), sweet cherry (*P. avium*) and apricot (*P. armeniaca*). In most cases SC has been

attributed to loss-of-pistil function or to mutations at the SFB gene. For example, in apricot and sweet cherry, the SC haplotypes are hypothesized to be a pollen-part mutant in most cases (Halasz et al, 2007; Marchese et al., 2007, Vilanova et al., 2006). However, the presence of a modifier locus outside the S-locus has also been reported in sweet cherry, almond and apricot (Cachi et al., 2014; Fernandez i Marti et al., 2009; Wünsch and Hormaza, 2004; Wünsch et al., 2010, Zuriaga et al., 2012., 2013). Other putative causes of SC found in Japanese plum and almond have been associated with a low transcriptional level in the S_e-RNase (Watari et al., 2007), or to epigenetic mutations in the S_f-RNase (Fernandez i Marti et al., 2014).

Whereas most work on GSI functioning in *Prunus* has focused on diploid species, several polyploid taxa are important fruit crops, including the tetraploid sour cherry (*P. cerasus*) and the hexaploid prune (*P. domestica*). Functioning of the SI system is likely to be considerably more complicated in polyploids than in diploids and appears to vary among taxonomic groups. For example, polyploidy in the Solanaceae induces a competitive interaction among alleles and the breakdown of the SI interaction with heteroallelic pollen (Golz et al 1999, 2001). Competitive interaction was reported by Huang et al (2008) for an SC selection of *P. pseudocerasus* that would be comparable with the Solanaceae. However, in *P. cerasus*, Hauck et al (2006) have shown that loss of SI is genotype dependent, rather than ploidy dependent, and that, in contrast to the Solananceae, heteroallelic pollen is SI. Detailed crossing studies between *P. cerasus* and the diploid *P. avium* support a one-allele match model, in which pollen is rejected when a functional *S*-haplotype occurs in both diploid pollen and tetraploid stylar tissue and so, breakdown of SI occurs when pollen contains two non-functional *S*-haplotypes that may be due to stylar, or pollen mutations (Hauck et al 2006, Tsukamoto et al. 2008).

Among polyploid *Prunus* species, relatively little is known about the GSI system in the hexaploid prune *Prunus domestica* L, including a dearth of information on allelic variation at the two loci within the S haplotype. This is surprising given the importance of prunes as functional foods with numerous beneficial health properties including, the prevention of cardiovascular disease, diabetes and obesity, and the promotion of digestive health (Stacewicz-Sapuntzakis, 2013). In addition, prunes have the unique capacity to prevent and to reverse bone loss associated with aging and estrogen deficiency (Wallace, 2017). Prune has become a critically important crop, with California the world leader in prune production. About 18 thousand hectares in California account for 99% of the U.S. supply and 40% of the world supply ($200 million annual production value). However, almost the entire California prune industry is a monoculture using the cultivar "Improved French". This makes the CA prune industry extremely vulnerable to catastrophic disease, pest outbreaks and periodic statewide crop failures due to adverse weather conditions such as high temperatures during the short bloom period. In addition, the entire prune crop must be harvested and dehydrated within a few weeks. To address these vulnerabilities, one of the main objectives of breeding programs worldwide is the development of new self-fertile cultivars with fruit that have low-moisture content and high nutritional values and that ripen sequentially to extend the harvest period of fruit during the summer months.

To date, only a few attempts have been made to identify *S*-allele sp[illegible]fic molecular markers for the identification of incompatibility cultivars in *P. domestica*. Lack of this information limits detailed characterization of germplasm, breeding efforts and appropriate orchard design. *S*-genotyping using consensus *S*-RNase primers developed in other *Prunus* species, [illegible]s successfully identified *S*-RNase alleles (Sutherland et al., 2004, 2008; Kota-Dombrovska and Lacis, 2013; Makovics and Halasz 2016; Abdallah et al., 2019). However, to date, only three *S*-RNase alleles have been cloned and sequences deposited in the NCBI repository (Sutherland et al., 2008), making genotyping analysis for the remaining alleles very difficult, since these are based on individual interpretations of banding patterns on gels.

In this study we use a multilevel approach that combines traditional field research with molecular gene analysis to understand variation in the *S*-RNase and SFB loci among cultivars and to ascertain the factors affecting reproductive success in *P. domestica*. This integrative approach (pollen tube growth, fruit set evaluation, PCR analysis, genomic DNA cloning/sequencing and 3D protein modelling) was undertaken in order to identify the SC haplotypes among thirty-six selections developed within the University of California, Davis prune breeding program and to characterize diversity at the *S* locus.

Material and methods

Plant material

Pistils and leaves from thirty-six different sel[illegible]tions and varieties, including Improved French, Sutter, Muir Beauty and Tulare Giant, were collected from adult trees growing in the UC Davis prune germplasm collection during three consecutive years (2018, 2019 and 2020). Name of the cultivars and breeding selections are listed in Table 1. Some breeding selections have been derived from crosses among Improved French prune, some others are imported prune clones from France and others are traditional European plum cultivars grown in the US. The UC Davis prune breeding program currently has around 75 selected candidate cultivars for potential release, as well as thousands of seedlings that require evaluation.

Pollen tube growth and fruit set

Twelve of the 36 selections formed an in vitro s[illegible]dy of self-pollination (Table 2). During three consecutive years (2018, 2019 and 202[illegible]), at least 12 flower buds a year, at stage D (Felipe 1977) from each of the 12 seedlings were collected from the field, emasculated in the laboratory and placed in a tray with tap-water, allowing the contact of the flower peduncles with the tray water to prevent dehydration. P[illegible]llen viability was determined by observing the germination of pollen grains in vitro. Anthers were removed from the emasculated flowers and allowed to dry for 48h. This pollen was used to self-pollinate the pistils in the tray. Ninety-six hours after pollination., the pistils were collected, placed in vials containing 5 ml of a 5% solution of Na_2SO_3 and autoclaved for 12 min at 1.2 kg cm^2

in order to soften the tissues. The samples were maintained at 4°C until observation., when they were stained with 0.1% (v/v) aniline blue in 0.1 N potassium phosphate as a specific stain for callose (Linskens and Esser, 1957). This growth was assessed by observation under an Olympus BH2 microscope with UV illumination with a Chiu Technical Corp with a Mercury-100 lamp. A genotype was considered SC when pollen tubes reached the base of at least 8 of the 12 styles, in a minimum of two years. In addition., a branch with a minimum of 100 flowers was bagged before bloom in the field., in order to assess the level of SC by evaluating fruit set in enclosed branches. Sets were calculated three months after bagging by counting the total number of fruits and were ranked according to Grasselly et al (1981), i) less than 2% = SI; ii) between 2% and 5% = low SC; iii) between 5% and 10% = SC and iv) higher than 10% = highly SC.

Isolation of genomic DNA

In addition to the twelve selections, total DNA from twenty-four more selections was isolated from young leaves using a Genomic DNA Purification Kit (Qiagen, USA). The quantification and quality evaluation of DNA was performed by a Qubit spectrophotometer (Thermo Fisher Scientific, USA).

S-locus fragment analysis

Pistil *S*-RNase genotyping was performed using four primer pairs that have shown good transferability among other *Prunus* species and have revealed high diversity within the *S*-RNase gene [PaConsIF/PaConsIIR and PaConsIF/EMPC5-consRD (Sonneveld et al., 2006; Sutherland et al., 2004), PruT2/PCER and PruC2/PCER (Tao et al., 1999; Yamane et al., 2001)]. For the pollen SFB gene, we used the primer combination Fbox5'F/FboxIntronR (Vaughan et al., 2006). All primer information can be found in Table 3.

PCR reactions for the *S*-RNase markers were performed in a volume of 25 μl containing 1x PCR buffer (Invitrogen), 1.5 mM $MgCl_2$, 0.2 mM dNTPs, 0.5 μM of each primer and 1 unit of Platinum® Taq DNA Polymerase (Invitrogen). The program consisted of a 2 min denaturation at 94ºC, then 35 cycles of 1 min at 94ºC, 2 min at 50ºC and 4 min at 68ºC followed by a final extension of 10 min at 68ºC. Amplification products were separated on 1% TAE agarose gel in 1X TAE buffer and stained with SYBRGreen and visualized with UV light. A 100 bp DNA ladder was used for fragment size determination.

PCR reaction for the SFB marker 'Fbox5'F/FboxIntronR' was performed with M13 labeled tail in its 5' extremity (CACGACGTTGTAAAACGACA). PCR conditions were as follow, 1X buffer containing 1.5 mM $MgCl_2$., 0.2 mM of each dNTP, 1U Taq polymerase (New England Biolabs), 0.02 μM forward primer with M13 tail in its 5´ extremity, 0.2 μM of M13 Dye (6-Fam) primer and 0.2 μM of reverse primer. The program consisted of 94 °C for 5 min; 30 cycles of 94 °C for 30 s., 56 °C for 45 s, and 72 °C for 45 s; 8 cycles of 94 °C for 30 s, 53 °C for 45 s, and 72 °C for 45 s; and a final elongation step at 72 °C for 10 min. PCR products were detected using an ABI PRISM 3730xl Genetic

Analyzer and GeneMapper analysis software (Applied Biosystems, CA, USA) located at the Evolutionary Genetics Lab (UC Berkeley).

Cloning, sequencing, primer design and analysis of the *S*-RNase alleles

Once the samples were genotyped, the different *S*-RNase alleles were gel-cut, cloned and sequenced. Prior to cloning, the band size corresponding to the target alleles was purified using the Wizard Plus Miniprep DNA Purification System (Promega, CA, USA) and quantified on 1.5% agarose gel using a standard 1 kb DNA ladder (Invitrogen, CA, USA). The purified PCR products were cloned into the vector pGEM using the Promega Cloning Kit. For each allele, at least three plasmids from different PCR reactions were sequenced from both ends following the methodology described by Fernandez i Marti et al (2010, 2014). Plasmids were sequenced on an automated sequencer ABI PRISM 3730 Genetic Analyzer (Applied Biosystems, CA). The coding sequences from C1 to C5 of the cloned *S*-alleles, were translated and the deduced amino acid sequences were aligned by the ClustalX method (Thompson et al., 1997) using MegAlign Software (DNASTAR., Madison, WI, USA). *S*-RNase sequences obtained in this work were deposited in GenBank under accession numbers MW407935- MW407936. In order to obtain a complete coverage of the *S*-RNase genes additional markers were developed by using the primer walking strategy. All primers were designed using the primer3 software (Koressaar et al., 2018). Specific primers for the S_{17} RNase allele were designed (Table 3). The PCR program consisted of an initial denaturation at 94 °C for 2 min, followed by 34 cycles of denaturation at 94 °C for 20 s, 40 s at 57 °C, extension at 72 °C for 1 min, and a final extension at 72 °C for 7 min. PCRs were set up on ice under sterile conditions and the thermocycler was preheated to 94 °C before adding the reactions. PCR products were run on a 1% agarose gel.

Diversity analysis and evolutionary phylogenetic analysis of *S* RNase alleles

P. domestica is believed to have originated as an interspecific hybrid of a diploid *P. cerasifera* and a tetraploid *P. spinosa*; the latter species may have been an interspecific hybrid of *P. cerasifera* and an unknown Eurasian plum species (Zhebentyayeva et al. 2019). The goal of our phylogenetic analysis was to investigate whether the origins of *S*-RNase alleles that we detected in *P. domestica* could be traced to its putative ancestors. Therefore, we obtained available GenBank sequences for 86 published SI alleles from members of subgenus *Prunus sensu* according to Shi et al (2013), see supplemental Table 1. Sequences included *S*-RNase alleles from the putative parents of *P. domestica* (*P. cerasifera* and *P. spinosa*) as well as the diploid plum (*P. salicina*) and seven randomly selected alleles for other *Prunus* species; *P. avium*, *P. dulcis* and *P. armeniaca*. Six *Pyrus communis* L. SI-RNase alleles were used as outgroup. Protein sequences for each species, together with the alleles detected in this study for *P. domestica*, were aligned using ClustalW integrated within the program Geneious. The alignment was visualized and manually refined using Jalview software (www.jalview.org). Phylogenetic analyses were performed using the maximum likelihood method through the customizable version of

RAxML 8.0 (Stamatakis, 2014). The resulting tree was visualized using FigTree v1.4.2 (http://tree.bio.ed.ac.uk/software/figtree/).

Three-dimensional Modelling of *S*-RNase proteins

The protein sequence of the *P. domestica* S_{17}RNase (MW407938.1; this work), along with protein sequences obtained from the NCBI database for the SC alleles [*P. dulcis* S_f-RNase (QDB64273.1), *P. armeniaca* S_c-RNase (ABO34168.1), *P. avium* $S_{6'}$-RNase (AAT72120.1), *P. salicina* S_e-RNase (BAF91848.1)] and the SI alleles [*P. domestica* S_{16}-RNase (MW407946.1), *P. cerasifera* S_1-RNase (AM992048.1) and *P. spinosa* S_{24}-RNase (ABV02077.1)] were used for 3D modelling analysis. The modelling procedure began with alignment of the sequences with the related known protein sequence and crystal structure (template) derived from the Protein Data Bank (PDB). The known 3D structure was chosen based on the protein sequence identity, which had to be higher than 35% with our target genes. Sequences were aligned using the deduced protein sequences for these *Prunus* RNase alleles using T-Coffee (EMBL-EBI). The frame of the 3D model was constructed by MODELLER 9v24 (Sali, 1993)). A total of 40 models were constructed for each *S*-RNase. The four models with the lowest value of the Modeller objective function were chosen for further refinement. Energy function was evaluated through PROSAIIv3 (Sippl 1993).

Stereo-chemical quality and the overall G-factors of the protein models were calculated using PROCHECK (Laskowski et al. 1993). The models with lower numbers of amino acid residues in disallowed regions were selected as the most suitable models. Finally., the molecular graphics of the best models were generated with PYMOL, which visualizes protein structures (http://www.pymol.org). Additionally, the 3D models of all S-RNases were compared with that of *Pyrus pyrifolia* S_3-RNase (BAA93052.1; Matsuura *et al.*, 2001), another Rosaceous species. Electrostatic and solvation properties were determined with the Adaptive Poisson-Boltzmann Solver84 using the PyMOL plug-in APBS Tool2.

Results

Phenotypic expression

In an attempt to characterize and identify the phenotypes associated with self-compatibility in *P. domestica*, we analyzed the pollen tube behavior in pistils under the microscope after self-pollinations. We confirmed *in vitro* tests by determining phenotypes of the same selections after bagging the branches in the field.

Observations of pollen tube growth allowed classifying the phenotype of the selections as SC or SI. Out of the twelve individuals analyzed in the lab, only seven (58.3%) showed pollen tubes at the base of the style (G16N10, G5N35, H13S-58, G16N19, Sutter, Muir Beauty and Improved French). As shown in Table 2, the percentage of pistils with pollen tubes reaching the ovary was between 76% (G16N19) and 91% (Muir Beauty). Consequently, these selections were considered as self-compatible. In the remaining five

samples, pollen tube growth was arrested in the middle third of the style of the self-pollinated pistils, and no pollen tubes were observed at the style base or in the ovary., displaying the characteristic arrest of pollen tube growth as the typical SI response (D6N-103, D18S-50, F3S-5, F13S-46 and Tulare Giant). Germinated pollen grains on the stigma were observed in all the selections.

Fruit set after bagging confirmed the SC/SI of all selections previously identified as SC/SI by pollen tube growth. Among the SC selections, five genotypes (G16N19, G5N35, Sutter, Muir Beauty and Improved French) were highly SC according to the criteria of Grasselly *et al*. (1981) with a fruit set higher than 10%, whereas the selections G16N19 and H13S-58 are considered as SC because fruit sets were between 5% and 10% (Table 2). In addition, sets after bagging confirmed the SI phenotype for the three years of the five individuals previously classified as SI under microscopic observation since their fruit set was lower than 2% in the three years.

Genotyping, cloning and sequencing analysis of the SI alleles in *P. domestica*

Of all primer combinations tested for the *S*-RNase locus, the primer set PruC2-PCER., which flanks the second and fifth conserved domains of *S*-RNases, was the most successful in all the samples. The number of alleles amplified with the other primer combinations was very low and, in most cases, no more than two alleles were identified for each individual. However, the PruC2-PCER primer set yielded a multiallelic profile showing from two (Muir Beauty) to five (WN16.1) different fragments per genotype. PCR allele identification revealed seventeen different bands on the gel, including one that was common to all SC phenotypes. This high number of amplified fragments reveals the high genetic diversity for *S* alleles in prune. Allele sizes ranged from 350 bp (S_{10}) to 1350 bp (S_{13}). The most common alleles were S_{17} that was common to all SC phenotypes and S_4, S_{14}, S_{12} and S_{11} which were observed in eight, or nine different selections (Figure 1). By contrast, allele S_{13} was only detected in one selection (Tulare Giant). A maximum of five alleles were found in one genotype (WN16.1). The average number of alleles per individual was three (Table 1).

We cloned and sequenced all bands amplified by primers PruC2-PCER to identify new alleles. Once all gel-band alleles were cloned and sequenced, a "blastn" analysis was conducted in order to find nucleotide sequence similarities with other *S*-alleles from *Prunus* species present in NCBI database. For all 17 alleles, we found high sequence similarities (≥95%) with S-RNase alleles from a range of species (Table 4).

PCR amplification of the intron of the pollen SFB revealed a total of fourteen different alleles within the thirty-five *P. domestica* genotypes for both primer combinations. The size obtained for Fbox5'F/Fbox–IntronR ranged between 192 bp and 375 bp, with the allele size 235 bp being the most common (found in 9 samples). By contrast, the SFB alleles with a size of 201, 207 and 240 were only detected in one selection.

A *S*-RNase allele common to self-compatible phenotypes of *P. domestica*

As described earlier, the S_{17} allele was present and common to all self-compatible phenotypes but was not detected in self-incompatible selections (Table 1). Sequences of three different cloned colonies for this band in multiple individuals of Improved French and Sutter showed a nucleotide match of 100%. Within the genus *Prunus*, the S_{17} allele from *P. domestica* showed 94% similarity with the S_5-RNase allele described for *P. virginiana* (JQ627793.1). We designed specific primers to amplify this allele. The new molecular marker was then used to detect presence/absence in the remainder of the selections. The primer pair produced a single band, with a size of 205 bp (Figure 2), in all genotypes that were classified as SC according to our phenotypic analyses. As expected, this new marker did not amplify any band in the SI genotypes.

Phylogenetic tree analysis and genetic relationship among the *S*-RNase alleles within the Rosaceae

We inferred evolutionary relationships among the *S*-RNase alleles of *P. domestica*, and among other *Prunus* species (*P. salicina*, *P. avium*, *P. dulcis*, *P. armeniaca*, *P. spinosa* and *P. cerasifera*), by constructing a ML phylogenetic tree of amino acid sequences (Figure 3). The *P. domestica S*-RNase alleles were widely dispersed in the ML phylogenetic tree, supporting previous reports of trans-specific origins. There was no evidence that *P. domestica S*-RNase-alleles clustered more frequently with *S*-RNase-alleles of putative hybrid parents, *P. spinosa* and *P. cerasifera*, than with alleles of any of the other *Prunus* species.

3D Modelling of S-RNase proteins

We constructed 3-D models of the SC-RNase alleles of *P. dulcis*, *P. armeniaca*, *P. avium* and *P. salicina* and compared these with the SI alleles of *P. domestica*, *P. cerasifera* and *P. spinosa* S_{24}-RNase as well as the *P. domestica* S_{17} based on related known 3D structures derived from the Protein Database (PDB). The best candidate template selected was the RNase MC1 mutant with accession 1J1G, with an identity between this template and the SC *P. domestica* S_f-RNase of 41% (E-value=3e^{-28}). The similarity to the SI *P. domestica* *S*-RNase was 40% and E-value=9e^{-32}. The MC1 protein sequence has 42% identity and E-value=4e^{-33}. The similarity to the S_e-RNase, S_6-RNase and S_c-RNase was 40%., 38% and 37.2%, respectively (E-value=3e^{-28}, E-value=9e^{-32} and E-value=1e^{-26}). The similarity and identity to *P. cerasifera* *S*-RNase and the *P. spinosa* S_{24}-RNase were 39% and 39.5%, with E-value=1e^{-29} and E-value=3e^{-37}.

Protein structures represent combinations of secondary structural elements., with α-helices from the core regions of the molecule connected by loop regions (β-sheets) on the protein surface. The structure of the *S*-RNases belonged to the α and β class, with six α helices and six β sheets connected by loops. The folding topologies of the main chains, found in the models analyzed here, were very similar to the topologies of the RNase T$_2$ family of enzymes. The MC1 protein sequence we used as a template has eight α-helices and eight β sheets, whereas protein structures generated for the SC *P. salicina*, *P. avium*, *P. spinos.*, *P.*

armeniaca and the S_{17} *P. domestica* alleles had 7 α-helices and between 2 and 6 β sheets (six for *P. salicina* and *P. spinosa*; four for *P. domestica* and *P. avium*; and two for *P. armeniaca*). The protein structure generated in *P. cerasifera*, SI *P. domestica* and SC *P. dulcis* S_f-RNase was 6 α-helices and 6 β sheets, with an overall molecular dimension for each *S*-RNase of approximately 40 Å x 50 Å x 30 Å.

Ramachandran plot statistics for the *S*-RNases showed that 98% of amino acid residues were positioned in the 'allowed' regions. Structures that place 95-97% or more of the amino acid residues in the 'allowed' positions., are considered to be reliable in modelling experiments, and this indicates how well the structures fit with the expected main chain length and torsion angle distributions. Thus, these results indicate that our models were optimal.

When all *S*-RNases were super positioned, the *P. domestica* S_{17}-RNase structure contained an additional extended loop., which was either not present or was much shorter than in the remaining SI *S*-RNases. When we aligned the amino acid sequences of the *P. domestica* S_{17}-RNase and the SI *P. domestica* *S*-RNase, we found that this long loop was associated with 17 amino acid residues (magenta loop in Figure 4) located between the conserved domains RC4 and C5 and corresponding to the sequence KRHSAQTKSGPKPLLLH. Zooming in on this 3D model structure revealed a very small loop found in the SI RNase allele in *P. domestica*, which had only 7 amino acids in that region of the gene (cyan loop; Figure 4).

Discussion

In this work, we report phenotypic expression of SI/SC and the isolation and characterization of novel *S*-RNase alleles in thirty-five genotypes of the hexaploid *P. domestica*. Also, we report on allelic variation within the SFB pollen part gene in the same selections of *P. domestica*.

Self-compatibility in *P. domestica*

To determine the status of self (in)compatibility in prunes, bagged branches and self-pollinations in the lab were conducted for three years. The phenotypes of the twelve selections were consistent using the two approaches. Pollen tube growth has been considered a clear indication of the compatibility of any pollination as it is independent of the environment where the study is done as in all cases the results have been unequivocal (Socias i Company et al., 2013). However, fruit set evaluation in the field is subject to many environmental hazards in spite of being the most natural approach to the real self-compatibility level of any genotype (Socias i Company et al., 2005). The differences between the results of different years point to unspecified environmental conditions affecting fruit set, stressing the need for self-compatibility evaluation in more than one year (Socias i Company et al., 2004). To our knowledge, this research represents the first study in *P. domestica* to evaluate self-compatibility for three years.

Pollen tube growth observations were robust during the three consecutive years, however,

the overall number of fruit set was slightly lower in 2020 than in 2018 and 2019. It has been reported in other species such as almond and tomato, that environment may strongly affect fruit set (Ganeva et al., 2019; Socias i Company et al., 2005). Although we observed a reduced fruit set in 2020, the sets obtained showed the ability of the flowers to set fruit with their own pollen.

Detection of *S*-RNase alleles

Self-incompatibility has been extensively studied in the most important diploid *Prunus* species, including Japanese plum (Watari et al., 2007), cherry (Tao et al., 1999; Wünsch and Hormaza, 2004), apricot (Vilanova et al., 2006), and almond (Fernandez i Marti et al., 2009). For initial *S* allele genotyping, we used SFB primers and consensus primers that amplified the *S*-RNase gene. These molecular markers of *S*-RNase and SFB loci have commonly been utilized to study the genetics of self-incompatibility in other *Prunus* species. For the *S*-RNase pistil component, we obtained reliable amplifications only with the PruC2-PCER primer combination that had been developed in sweet cherry (Tao et al, 1999). Similar results were described by Abdallah et al. (2019), where, out of all the primers tested, the PruC2-PCER primer combination was the most successful. Some of the other conserved primers were developed in almond, subgenus *Amygdalus*, suggesting possible low transferability to the subgenus *Prunus*. Although Sutherland et al. (2008) suggested that primers spanning the 2nd intron, such as PruC2, were not very informative in *P. domestica* due to a narrow range of amplification fragments, we were able to amplify up to five different fragments per sample. Our results agreed with previous *S*-genotyping analyses which revealed a high number of amplified fragments in prune by using the same primer combination (Kota and Lacis, 2013).

We corroborated our *S*-genotyping results with phenotypical evaluations in the field and observations of pollen tube growth in pistils after self-pollinations under the microscope. In total, we detected seventeen different *S*-RNase alleles, one of which was common and exclusive to all SC phenotypes among the thirty-six prune cultivars. The alleles we detected included the RNase of the three haplotypes (S_5, S_6 and S_9) reported by Sutherland et al. (2008). The most frequent alleles among our tested selections were S_4, S_{11}, S_{12}, S_{14} and S_{17} which, based on the blast analysis, had high similarities with alleles described in *P. salicina*, *P. persica* and *P. virginiana*. Although the similarity of our alleles was high when compared to other species (between 95% and 98%), we observed several indels and nucleotide differences. Thus, our results suggest that they can be considered as new alleles within the Rosaceae. Only the three alleles reported in *P. domestica* by Sutherland et al (2008) plus the alleles S_1 and S_2 that were 100% identical to S_{17}RNase and S_{27}RNase in *P. salicina*, have been previously published in the NCBI GenBank.

Self-compatibility in *P. domestica*

Approximately one third of the total cultivars and selections we analyzed (31.5%) were self-compatible. This genotypic variation in SI/SC expression suggests that the observation by Hauck et al (2006) that polyploidy *per se* is not the cause of SC in the tetraploid *P. cerasus* can equally be applied to the hexaploid *P. domestica*. Some of our selections have

their same origin in Improved French, which is well known to be a SC variety and so the expression of SC in our selections could be associated with inheritance of the determinants in this cultivar. The breakdown of SI in Improved French has not been investigated and could have several origins, including stylar *S* RNase, and pollen SFB mutations, or factors external to the *S*-locus. We found a 300bp fragment of the SFB intron that was associated with the SC selections and correlated with the S_{17} RNase allele. Therefore, we cannot speculate whether the loss of SI among our selections of *P. domestica* may be caused by stylar or pollen mutations. Mutations in the SFB gene appear to be most common in SC diploid *Prunus* species e.g. *P. armeniaca* (Vilanova et al., 2006; Halasz et al., 2007), *P. mume* (Tao et al., 2007; Ushijima et al., 2004) and *P. avium* (Ushijima et al., 2004; Sonnelveld et al., 2005; Marchese et al., 2007) whereas both stylar and pollen part mutations have been reported to cause breakdown of SI in the polyploid *P. cerasus* (Hauck et al 2006, Tsukamoto 2006, 2008). SC may also be determined by a factor external to the *S* locus. This was suggested by Cachi and Wünsch (2011) for the variety Cristobalina of *P. avium* and recent molecular studies have detected a putative modifier region (Ono et al 2018). Interestingly, in Cristobalina, self-fertilized pollen tube growth is slower than cross-fertilized, suggesting a form of partial SC (Cachi et al. 2014). A modifier gene mediating the pollen part conferring SC has also been detected in *Prunus armeniaca* (Zuriaga et al. 2012, 2013) and in *Prunus dulcis*, where some genotypes lacking the SC allele had a self-compatible phenotype in the field (Fernandez i Marti et al., 2009).

S-RNase relationships among *Prunus* species including putative parents of hexaploid *P. domestica*

The sequence differences that we detected at the *S*-RNase locus agree with the hypothesis that new S-alleles may be generated by the accumulation of point mutations within the female component (Despres et al., 1994). These genetic variations can arise from gene mutations, or from genetic recombination near introns, as suggested by Ortega et al. (2006), leading to alterations in gene activity or protein function. We found high sequence similarity (≥ 95%) for S-RNase alleles with a range of species in the genus *Prunus*, suggesting orthologous alleles. The ML phylogenetic tree showed no evidence that alleles detected in *P. domestica* were more closely associated with those of the putative parents, *P. spinosa* and *P cerasifera* than with any other of the *Prunus* species studied. The *P. domestica* alleles were spread across the phylogeny, consistent with trans-specific origins described by Richman and Kohn (2000) and others (Tsukamoto 2008), although, as pointed out by Vieira et al (2008), the allele origins in *Prunus* are not as ancient as in some other groups, such as the Solanaceae.

3-D structure of S-RNase alleles

We used PYMOL to visualize the 3-D structure of amino acid residues within the *S*-RNase gene. It has been suggested that loops in 3D structures serve to interconnect α-helices and β-strands, and that longer surface-exposed loops could be susceptible to proteolytic degradation (Branden and Tooze 1998). The general functional differences among 3D almond SC and SI RNases involved an extended loop in the S_f-RNase (Fernandez i Marti et al., 2010; Goonetilleke et al., 2020). Furthermore, the main structural difference found

in the three-dimensional models generated in this study between the SC and SI *P. domestica* proteins resided in the presence and length of the loop within the conserved domains RC4 and C5. When we analyzed the modeled SC proteins for the S_e-RNase of *P. salicina*, $S_{6'}$-RNase of *P. avium* and S_c-RNase of *P. armeniaca*, the three 3D structures also presented the same loop pattern and were similar in length to the SC *P. domestica*. The protein structure of the SI RNases of *P. spinosa* and *P. cerasifera* either lacked this loop., or the loop was shorter than that of the SC alleles in *P. avium*, *P. armeniaca*, *P. dulcis* or *P. domestica*. Variation in the number, lengths, and positions of α-helices, β sheets and loops seems to contribute to functional differences among *S*-RNases in *Prunus*. Although it is unknown if this long loop has a direct effect on autogamy in plants, it is interesting that all the SC *S*-RNases in *Prunus* share this unique feature.

Conclusion

This study describes for the first time a large number of S-RNase alleles in the hexaploid *P. domestica*. *S*-genotyping, cloning and sequencing allowed us to identify and characterize 17 *S*-RNase alleles, of which 13 are considered new and can be used for orchard design and selection of parental genotypes in prune breeding programs. In addition, our study describes for the first time the association and design of a molecular marker for self-compatibility in *P. domestica*. Our phylogenetic tree showed that the *S*-alleles, are spread across the phylogeny, suggesting that they were of trans-specific origin. Three-dimensional *S*-RNase structures were analyzed for the first time in plum, cherry, apricot as well as in *P. cerasifera* and *P. spinosa*.

Acknowledgements

This research was supported, in part., by the California Prune Board. AFM wants to dedicate this article to Dr. Socias i Company, who passed away last November 2020. Dr. Socias was one of the pioneers to discover the trait of SI in fruit trees, almost 50 years ago, and it is from him that AFM inherited this passion for working in tree breeding and genetics.

References

Abdallah D, Baraket G, Perez V, Ben Mustapha S, Salhi Hannachi A, Hormaza I. Analysis of self-incompatibility and genetic diversity in diploid and hexaploid plum genotypes. Front Plant Sci, 2019. https//doiorg/103389/fpls201900896.

Akagi T, Henry IM, Morimoto T, Tao R. Insights into the *Prunus*-specific S-RNase-based self-incompatibility system from a genome-wide analysis of the evolutionary radiation of *S* locus-related F-box genes. Plant Cell Physiol. 2016. 57: 1281–1294.

Cachi AM, Wünsch A. Characterization and mapping of non-S gametophytic self-compatibility in sweet cherry Prunus avium L. J Exp Bot.2011. 62: 1847–1856.

Cachi AM, Hedhly A, Hormza JI, Wünsch A. Pollen tube growth in the self-compatible sweet cherry genotype 'Cristobalina' is slowed down after self-pollination. Ann Appl Biol.2014. 164: 73–84.

de Nettancourt D. Incompatibility in angiosperms. Springer-Verlag Berlin Heidelberg New York.1977.

Després C, Saba-El-Leil M, Rivard SR, Morse D, and Cappadocia M. Molecular cloning of two Solanum chacoense S-alleles and a hypothesis concerning their evolution. Sexual Plant Reproduction. 1994 . 7: 169–176.

Entani T, Iwano, M Shiba H, Che FS, Isogai A, Takayama S. Comparative analysis of the self-incompatibility *S*-locus region of *Prunus mune* Identification of a pollen-expressed F-box gene with allelic diversity. Genes Cells. 2003 . 8: 203-213.

Felipe AJ. Almendro estados fenológicos. Inf Técn Econ Agrar.1977. 27: 8-9.

Fernández i Martí A Hanada T Alonso JM Yamane H Tao R Socias i Company R. A modifier locus affecting the expression of the *S*-RNase gene could be the cause of breakdown of self-incompatibility in almond. Sex Plant Reprod. 2009. 22: 179-186.

Fernández i Martí A, Hanada T, Alonso JM, Yamane H, Tao R, Socias i Company R. The almond S_f haplotype shows a double expression despite its comprehensive genetic identity. Scientia Hortic. 2010. 125: 685-691.

Fernández i Martí A, Gradziel T, and Socias i Company R. Methylation of the S_f allele in almond is associated with S-RNase loss of function. Plant Mol Biol. 2014. 86: 681–689.

Ganeva D, Grozeva S, and Pevicharova G. Effect of reduced irrigation on flowering fruit set and yield of indeterminate tomato. International Journal of Recent Technology and Engineering. 2014. 8: 2277-3878.

Grasselly C, Crossa-Raynaud P, Olivier G, Gall H. Transmission du caractère d'autocompatibilité chez l'amandier *Amygdalus communis*. Options Méditerr CIHEAM/IAMZ. 1981. 81:I71-75.

Goldraij A, Kondo K, Lee CB, Hancock CN, Sivaguru M, Vazquez-Santana S, Kim S, Phillips TE, Cruz-Garcia F, McClure B. Compartmentalization of *S*-RNase and HT-B degradation in self-incompatible *Nicotiana*. Nature. 2006. 439: 805-810.

Goonetilleke SN, Croxford AE, March T, Wirthensohn M, Hrmova M, and Diane E. Variation among S-locus haplotypes and among stylar RNases in almond. Scientific Reports. 2020. 10: 583.

Gu Chao, Wang L, Korban S, and Han Y. Identification and characterization of S-RNase genes and S-genotypes in Prunus and Malus species. Can J Plant Sci. 2015. 95: 1–13.

Hafizi A, Shiran B, Maleki B, Imani A, and Banović B. Identification of new S-RNase self-incompatibility alleles and characterization of natural mutations in Iranian almond cultivars. Trees.2013. 27: 497–510.

Halász J, Pedryc A, and Hegedűs A. Origin and dissemination of the pollen-part mutated S C-haplotype that confers self-compatibility in apricot Prunus armeniaca. New Phytol. 2007. 176: 793-803.

Hauck NR, Yamane H, Tao R, Iezzoni AF. Accumulation of nonfunctional S-haplotypes results in the breakdown of gametophytic self-incompatibility in tetraploid *Prunus*. Genetics. 2006. 172: 1191-1198.

Huang S-X, Wu H-Q, Li Y-R, Wu J, S-J Heng W, and Zhang S-L. Competitive interaction between two functional S-haplotypes confer self-compatibility on tetraploid Chinese cherry *Prunus pseudocerasus* LindlCV Nanjing Chuisi. Plant Cell Reports. 2008. 27:1075–1085.

Igic B, and Kohn JR. Evolutionary relationships among self-incompatibility RNases. Proc Natl Acad Sci USA. 2001. 98: 13167-13171.

Ikeda K, Ushijima K, Yamane H, Tao R, Hauck NR, Sebolt AM, and Iezzoni. AF. Linkage and physical distances between the S-haplotype S-RNase and SFB genes in sweet cherry. Sex Plant Reproduction. 2005. 17: 289-296.

Kao TH, and Tsukamoto T. The molecular and genetic bases of S-RNase-based self-incompatibility. Plant Cell. 2004. 16: 72–3.

Koressaar T, Lepamets M, Kaplinski L, Raime K, Andreson R, and Remm M. Primer3 marker integrating masking of template sequence with primer design software. Bioinformatics. 2018. 3411: 1937-1938.

Kota-Dombrovska I, and Lâcis G. Evaluation of self-incompatibility locus diversity of domestic plum Prunus domestica L using DNA-based S-genotyping. Proc Latv Acad Sci B. 2013. 672-683: 109–115.

Kubo K, Paape T, Hatakeyama M, Entani T, Takara A, Kajihara K. et al. Gene duplication and genetic exchange drive the evolution of *S*-RNase-based self-incompatibility in *Petunia*. Nat Plants. 2015. 1: 14005.

Laskowski RA, MacArthur MW, Moss DS, Thornton JM. PROCHECK—a program to check the stereochemical quality of protein structures. J Applied Crystallogr. 1993. 26: 283–91.

Makovics-Zsohár N, and Halász J. Self-incompatibility system in polyploid fruit tree species- A review. The International Journal of Plant Reproductive Biology. 2016. 8; 1.

Marchese A, Boskovic RI, Caruso T, Raimondo A, Cutuli M, and Tobutt KR. A new self-compatibility haplotype in the sweet cherry 'Kronio' S5' attributable to a pollen-part mutation in the SFB gene. J Exp Bot. 2007. 58: 4347–4356.

Matsumoto D, and Tao R. Recognition of a wide-range of S-RNases by S locus F-box like 2 a general-inhibitor candidate in the *Prunus*-specific S-RNase-based self-incompatibility system. Plant Mol Biol. 2016. 91: 459–469.

McClure BA, Mou B. Canevascini S. and Bernatzky R. A small asparagine-rich protein required for S-allele specific pollen rejection in Nicotiana. Proc Natl Sci USA. 1999. 96:13548-13553.

Muñoz-Sanz JV, Zuriaga E, López I, and Badenes ML. Romero C. Self-in compatibility in apricot germplasm is controlled by two major loci *S* and *M*. BMC Plant Biology. 2017. 17:82.

Ono K, Akagi T, Morimoto T, Wünsch A, Tao R. Genome Re-Sequencing of Diverse Sweet Cherry Prunus avium Individuals Reveals a Modifier Gene Mutation Conferring Pollen-Part Self-Compatibility. *Plant and Cell Physiology*. 2018. 59: 1265–1275.

Ortega E, Bošković R, Sargent DJ, Tobutt KR. Analysis of *S*-RNase alleles of almond *Prunus dulcis* characterization of new sequences resolution of synonyms and evidence of intragenic recombination. Mol Gen Genomics. 2006. 276: 413-426.

Richman AD, and Kohn JR. Evolutionary genetics of self-incompatibility in the Solanaceae. Plant Molecular Biology. 2000. 42: 169–179.

Šali A. and Blundell TL. Comparative protein modelling by satisfaction of spatial restraints. J Mol Biol. 199. 234: 779-81.

Sassa H, Hirano H, Nishio T, and Koba T. Style-specific self-incompatibility mutation caused by deletion of the *S*-RNase gene in Japanese pear *Pyrus serotina*. Plant J.1997. 12: 223–227.

Shi S, Li J, Sun J, Yu J, and Zhou S. Phylogeny and classification of *Prunus sensu lato* Rosaceae. J Integr Plant Biol. 2013. 55: 1069–1079.

Sippl MJ. Recognition of errors in three-dimensional structures of proteins. Proteins. 1993. 17: 355-62.

Socias i Company R, Alonso JM, and Gómez-Aparisi J. Fruit set and productivity in almond as related to self-compatibility flower morphology and bud density. J Hort Sci Biotechnol. 2004. 79: 754-758.

Socias i Company R, Gómez Aparisi J, and Alonso JM. Year and enclosure effects on fruit set in an autogamous almond. Scientia Horticulturae. 2005. 104: 369-377.

Stacewicz-Sapuntzakis M. Dried plums and their products composition and health effects– an updated review. Crit Rev Food Sci Nutr. 2013. 53: 1277–1302.

Stamatakis A. RAxML version 8 a tool for phylogenetic analysis and post-analysis of large phylogenies. Bioinformatics. 2014. 30: 1312–1313.

Sutherland BG, Robbins TP, and Tobutt KR. Primers amplifying a range of *Prunus* S-alleles. Plant Breed. 2004. 123: 582-584.

Sutherland G, Tobutt R, and Robbins P. Trans-specific *S*-RNase and SFB alleles in *Prunus* self-incompatibility haplotypes. Mol Genet Genomics. 2008. 279: 95–106.

Tao R, Yamane H, Sassa H, Mori H, Gradziel TM, Dandekar AM, and Sugiura A. Identification of stylar RNases associated with gametophytic self-incompatibility in almond *Prunus* dulcis. Plant Cell Physiol. 1997. 38: 304-311.

Tao R, Yamane H, Sugiura A, Murayama H, Sassa H, et al. Molecular typing of S-alleles through identification characterization and cDNA cloning for S-RNases in sweet cherry. J Am Soc Hortic Sci1999. 124: 224–233.

Thompson JD, Gibson T, Plewniak F, Jeanmougin F, and Higgins D. The CLUSTAL_X windows interface flexible strategies for multiple sequence alignment aided by quality analysis tools. Nucleic Acids Res. 1997. 25: 4876–4882.

Tsukamoto T, Hauck NR, Tao R, Jiang N, and Iezzoni AF. Molecular characterization of three non-functional S-haplotypes in sour cherry Prunus cerasus. Plant Molecular Biology2006. 62: 371.

Tsukamoto T, Potter D, Tao R, Vieira J, and Iezzoni AF. Genetic and molecular characterization of three novel S-haplotypes in sour cherry Prunus cerasus L. Journal of Experimental Botany2008. 59: 3169-3185.

Ushijima K, Sassa H, Dandekar MA, Gradziel TM, Tao R, Hirano H. Structural and transcriptional analysis of the self-incompatibility locus of almond identification of a pollen-expressed F-box gene with haplotype-specific polymorphism. Plant Cell. 2003. 15:771-781.

Ushijima K, Sassa H, Tao R, Yamane H, Dandekar MA, Gradziel TM, and Hirano H. Cloning and characterization of cDNAs encoding S-RNases from almond *Prunus dulcis* primary structural features and sequence diversity of the S-RNases in Rosaceae. Mol Gen Genet. 1998. 260: 261-268.

Vaughan SP, Russell K, Sargent DJ, and Tobutt KR. Isolation of S-locus F-box alleles in *Prunus avium* and their application in a novel method to determine self-incompatibility genotype. Theor Appl Genet. 2006. 112: 856-66.

Vieira J, Fonseca NA, Santos RM, Habu T, Tao R, and Vieira CP. The number age sharing and relatedness of S-locus specificities in *Prunu.s* Genet Res. 2008. 90: 17–26.
Vilanova S, Badenes ML, Burgos L, Martínez-Calvo J, Llácer G, and Romero C. Self-compatibility of two apricot selections is associated with two pollen part mutations of different nature. Plant Physiol. 2006. 142: 629-641.
Wallace TC. Dried plums prunes and bone health a comprehensive review. Nutrients. 2017. 9 E401.
Watari A, Hanada T, Yamane H, Esumi T, Tao R, Yaegaki H et al. A low transcriptional level of Se-RNase in the Se-haplotype confers self-compatibility in Japanese plum. *J Am Soc Hort Sci*. 2007. 132: 396–406.
Wünsch A, and Hormaza JI. Genetic and molecular analysis in Cristobalina sweet cherry a spontaneous self-compatible mutant. Sex Plant Reprod. 2004. 17: 203–210.
Wünsch A, Tao R, and Hormaza JI. Self-compatibility in 'Cristobalina' sweet cherry is not associated with duplications or modified transcription levels of S-locus genes. Plant Cell report. 2010. 29: 715-721.
Xu G, Ma H, Nei M, and Kong H. Evolution of F-box genes in plants Different modes of sequence divergence and their relationships with functional diversification. PNAS. 2009. 106: 835-840.
Yamane H, and Tao R. Molecular basis of self-incompatibility and current status of S-genotyping in rosaceous fruit trees. J Jpn Soc Hortic Sci. 2009. 78:137–157.
Zhebentyayeva T, Shankar V, Scorza R *et al*. Genetic characterization of worldwide *Prunus domestica* plum germplasm using sequence-based genotyping. Hortic Res. 2019. **6**: 12.
Zuriaga E, Molina L, Badenes ML, Romero C. Physical mapping of a pollen modifier locus controlling self-incompatibility in apricot and synteny analysis within the Rosaceae. Plant Mol Biol. 2012. 79: 229–242.
Zuriaga E, Munoz-Sanz JV, Molina L, Gisbert AD, Badenes ML, Romero C. An S-locus independent pollen factor confers self-compatibility in 'Katy' apricot. PLoS One. 2013. 8: e53947.

Figure captions

Figure 1, PCR amplification of 36 dried prune cultivars and selections, obtained with the primers PruC2-PCER. Assessment of the alleles was accomplished after individual band isolation, cloning and sequencing.

Figure 2, Example of efficacy of the new S_{17} marker tested in three SC and three SI samples. Improved French (1), Sutter (2), Muir Beauty (3), Tulare Giant (4), 18S-50 (5) and 6N-103 (6).

Figure 3, Phylogenetic relationships among S-RNase alleles from Prunus species including, the 17 alleles identified in this study. Pyrus was used as an outgroup.

Figure 4, Stereo representation (a) and ribbon diagram (b) of modeled structure of *P. domestica* S_{17}-RNase (magenta), and *P. domestica* SI-RNase (cyan), showing secondary structural elements and surfaces.

Table 1, Summary of the 36 selections of *P. domestica* used in this study, including their origins and their detected *S*-RNase-genotypeS

Sample	Name	Pedigree	Detected S-genotype	SC/SI
1	F3S-5	Sutter OP	S1/S5/S14	SI
2	F11S-38	French X 6-22-51	S4/S17	SC
3	G5N-35	French X Sutter	S12/S14/S17	SC
4	G45N-35	D6N103 X Muir Beauty	S8/S14/S16	SI
5	G3N-16	S19-39 X 6-22-51	S5/S7/S11/S14	SI
6	D18S-50	Imperial X Tulare Giant	S11/S12/S16	SI
7	G16N-19	3-9E-49 X 4-7E-35	S11/S14/S17	SC
8	G40N-34	D2N-26 X Muir Beauty	S4/S7	SI
9	2-2E-38RR	Early Tragedy X Primacotes	S5/S7/S9/S16	SI
10	D3-39	French OP	S6/S17	SC
11	3-11E-45RR	French X Tardicotes	S6/S9/S12	SI
12	3-11E-32RR	French X Tardicotes	S2/S3/S4/S12	SI
13	5/1/32	French OP	S2/S4/S7/S12	SI
14	3-8E-46RR	French X Tragedy	S1/S3/S6/S17	SC
15	Tragedy	German Prune X Purple Twain	S7/S12	SI
16	Fruit Salad	--	S3/S6	SI
17	Improved French	Agen X seedling of Agen	S10/S12/S17	SC
18	Sutter	Sugar X Primacote	S3/S10/S12/S17	SC
19	F9N-21	Tulare Giant X 6-22-52	S3/S6	SI
20	3-9E-49	--	S3/S9/S11/S17	SC
21	F11N-34	5-3-4 X X 6-19-51	S4/S6	SI
22	G43N-1	D4N-46 X Muir Beauty	S5/S7/S10/S12	SI
23	F13N-23	French X 6-22-51	S4/S9/S10	SI
24	Jojo	Ortenauer X Stanley	S1/S3/S5/S10	SI
25	F2N-10	5-19-30 OP	S1/S9/S14/S15	SI
26	G5N-58	French X Sutter	S7/S11/S12	SI
27	Tulare Giant	Empress X Primacote	S7/S13/S16	SI
28	D6N-103	French X 5-1-32	S7/S8/S12	SI
29	F9S-33	Tulare Giant X 6-22-51	S8/S9/S15	SI
30	Muir Beauty	French X Tulare Giant	S10/S17	SC
31	F13S-46	French X 6-22-51	S4/S10/S11/S15	SI
32	G2N-24	5-19-39 X 6-15-62	S4/S15	SI
33	29C	Rootstock	S6/S11/S14/S17	SC
34	WS3.1	--	S2/S4/S9/S15	SI
35	WN16.1	--	S7/S8/S12/S15/S16	SI

772	36	H13S-58	D2N-76 OP	S6/S11/S14/S17	SC

Table 2, Data showing fruit set after bagging and pollen tube growth for three years and phenotype for SC/SI from the evaluations in the field and in the lab under microscope observations.

Selection	Fruit Set % 2018	Fruit Set % 2019	Fruit Set % 2020	Average. Fruit Set %	Phenotype Fruit set	Number of analyzed pistils	% Pistils with pollen tubes reaching the ovary	Phenotype microscope
Improved French	26.7	25.2	21.1	24.3	Highly SC	38	87	SC
Sutter	12	16	14	14.0	Highly SC	40	85	SC
Muir Beauty	32	30.1	25.4	29.2	Highly SC	36	91	SC
G5N35	19	25	21	21.7	Highly SC	35	87	SC
G16N19	31	28	23	27.3	Highly SC	38	89	SC
H13S-58	7.2	6.2	5.1	6.2	SC	36	77	SC
G16N19	11	8.6	7	8.9	SC	37	76	SC
D6N-103	0.9	1.6	0	0.8	SI	37	0	SI
D18S-50	0	0	0	0.0	SI	39	0	SI
F3S-5	0.7	1.5	1.2	1.1	SI	36	0	SI
F13S-46	0	0	0	0.0	SI	36	0	SI
Tulare Giant	1	0.6	0	0.5	SI	38	0	SI

Table 3, Sequence information of the primers used and designed in this study.

**Primer name**	**Sequence 5' 3'**	**Reference**
PaConsIF	MCTTGTTCTTGSTTTYGCTTT CTTC	Sonneveld et al., 2006
PaConsIIR	CAWAACAAARTACCACTTCATGTAAC	Sonneveld et al., 2006
EMPC5-consRD	CAAAATACCACTTCATGTAACARC	Sutherland et al., 2004
PruT2	TSTTSTTGSTTTTGCTTTCTTC	Tao et al., 1999
PCER	TGTTTGTTCCATTCGCYTTCCC	Tao et al., 1999
PruC2	CTATGGCCAAGTAATTATTCAAACC	Yamane et al., 2001
S17-F*	TCTTCCCTTGCTTGGTGTCT	*This study
S17-R*	TCCATGTCTGTGTCGGATGT	*This study
Fbox5'F	TTKSCHATTRYCAACCKCAAAAG	Vaughan et al., 2006
FboxIntronR	CWGGTAGTCTTDSYAGGATG	Vaughan et al., 2006

Table 4, Size in base pairs (bp) of the new *S* alleles discovered in this study for RNase and SFB genes and sequence similarities with *S*-RNase alleles from a range of species in the genus *Prunus*

Allele this study	Orthologous allele	Species with orthologous gene	% similarity	PRUC2-PCER (bp)	Fbox5'F-FboxIntronR (bp)
S_1	S_{17}RNase	*P. salicina*	100	780	375
S_2	S_{27}RNase	*P. salicina*	100	380	n.a
S_3	S_6RNase	*P. domestica*	96	615	n.a
S_4	S_1RNase	*P. persica*	98	585	230
S_5	S_5RNase	*P. domestica*	100	695	235
S_6	S_6RNase	*P. domestica*	100	310	274
S_7	S_4RNase	*P. cerasifera*	99	745	201
S_8	Unidentified	*P. cerasifera*	99	1250	n.a
S_9	S_9RNase	*P. domestica*	100	470	365
S_{10}	S_{10}RNase	*P. cerasifera*	97	350	196
S_{11}	S_{24}RNase	*P. salicina*	98	*525*	*240*
S_{12}	S_{18}RNase	*P. virginiana*	97	805	192
S_{13}	S_{31}RNase	*P. speciosa*	95	1350	225
S_{14}	S_{12}RNase	*P. virginiana*	95	365	215
S_{15}	S_{24}RNase	*P. spinosa*	98	710	207
S_{16}	S_8RNase	*P. spinosa*	95	890	242
S_{17}	S_5RNase	*P. virginiana*	94	395	300

n.a, no amplification.

Supplementary Table 1, *S* alleles, specie name and accession number for each sequence obtained from NCBI used for the construction of the phylogenetic tree in Figure 3.

**S allele**	**Species**	**NCBI code**
S1	*Prunus domestica*	MW407936.1
S2	*Prunus domestica*	EU113267.1
S3	*Prunus domestica*	MW407937.1
S4	*Prunus domestica*	MW407939.1
S5	*Prunus domestica*	MW407935.1
S6	*Prunus domestica*	AM746947.1
S7	*Prunus domestica*	AM992050.1
S8	*Prunus domestica*	CAQ51498.1
S9	*Prunus domestica*	AM746948.1
S10	*Prunus domestica*	MW407940.1
S11	*Prunus domestica*	MW407941.1
S12	*Prunus domestica*	MW407942.1
S13	*Prunus domestica*	MW407943.1
S14	*Prunus domestica*	MW407944.1
S15	*Prunus domestica*	MW407945.1
S16	*Prunus domestica*	MW407946.1
S17	*Prunus domestica*	MW407938.1
S1	*Prunus cerasifera*	AM992048.1
S2	*Prunus cerasifera*	AM992049.1
S3	*Prunus cerasifera*	AM746943.1
S4	*Prunus cerasifera*	AM992050.1
S5	*Prunus cerasifera*	AM992051.1
S6	*Prunus cerasifera*	AM992052.1
S7	*Prunus cerasifera*	AM992053.1
S8	*Prunus cerasifera*	AM992054.1
S9	*Prunus cerasifera*	AM746944.1
S10	*Prunus cerasifera*	AM746945.1
S11	*Prunus cerasifera*	AM992055.1
S12	*Prunus cerasifera*	AM992056.1
S13	*Prunus cerasifera*	AM992057.1
S14	*Prunus cerasifera*	AM992058.1
Unknown_M4	*Prunus cerasifera*	CAQ51498.1
S1	*Prunus spinosa*	EF636467.1
S3	*Prunus spinosa*	DQ677584.1
S7	*Prunus spinosa*	DQ677586.1
S8	*Prunus spinosa*	DQ677587.1

S9	*Prunus spinosa*	DQ677588.1
S10	*Prunus spinosa*	DQ677589.1
S12	*Prunus spinosa*	DQ677590.1
S15	*Prunus spinosa*	EF636468.1
S16	*Prunus spinosa*	EF636469.1
S19	*Prunus spinosa*	EF636470.1
S24	*Prunus spinosa*	EF636471.1
S5	*Prunus virginiana*	JQ627793.1
S12	*Prunus virginiana*	JQ627800.1
S18	*Prunus virginiana*	JQ627806.1
S13	*Prunus armeniaca*	EF062341.1
S17	*Prunus armeniaca*	EU516388.1
S19	*Prunus armeniaca*	EF133689.1
S24	*Prunus armeniaca*	HQ615602.1
S40	*Prunus armeniaca*	GU354239.1
S52	*Prunus armeniaca*	KF951503.2
Sc	*Prunus armeniaca*	ABO34168.1
S3	*Prunus dulcis*	KY059853.1
S10	*Prunus dulcis*	AM231658.1
S11	*Prunus dulcis*	AM231660.1
S15	*Prunus dulcis*	AM231664.1
S21	*Prunus dulcis*	EF690369.1
S23	*Prunus dulcis*	FN429354.1
Sf	*Prunus dulcis*	AB467371
S9	*Pyrus pyrifolia*	AB104909.1
S10	*Pyrus pyrifolia*	AY158069.1
S15	*Pyrus pyrifolia*	AY249430.2
S18	*Pyrus pyrifolia*	KY608870.1
S34	*Pyrus pyrifolia*	DQ269500.1
S44	*Pyrus pyrifolia*	KY608875.1
S4sm	*Pyrus pyrifolia*	AB009385.1
S1	*Prunus avium*	AY851457.1
S5	*Prunus avium*	AJ298314.1
S6'	*Prunus avium*	AAT72120.1
S12	*Prunus avium*	AY259115.1
S18	*Prunus avium*	DQ508941.1
S21	*Prunus avium*	DQ508944.1
S38	*Prunus avium*	JQ280516.1
S1	*Prunus salicina*	AF433649.1
S3	*Prunus salicina*	AF432417.1

S4	*Prunus salicina*	AF432418.1
S5	*Prunus salicina*	AF433647.1
S6	*Prunus salicina*	AF433648.1
S7	*Prunus salicina*	AY781290.1
S8	*Prunus salicina*	AY902455.1
S9	*Prunus salicina*	AY996051.1
S10	*Prunus salicina*	DQ003310.1
S11	*Prunus salicina*	DQ512908.1
S12	*Prunus salicina*	DQ512909.1
S15	*Prunus salicina*	EF177346.1
S16	*Prunus salicina*	EF177347.1
S17	*Prunus salicina*	EF177348.1
S18	*Prunus salicina*	EF177349.1
S19	*Prunus salicina*	EU113259.1
S20	*Prunus salicina*	EU113260.1
S21	*Prunus salicina*	EU113261.1
S22	*Prunus salicina*	EU113262.1
S23	*Prunus salicina*	EU113263.1
S24	*Prunus salicina*	EU113264.1
S25	*Prunus salicina*	EU113265.1
S26	*Prunus salicina*	EU113266.1
S27	*Prunus salicina*	EU113267.1
S32	*Prunus salicina*	GU574195.1
Se	*Prunus salicina*	BAF91848.1
S41	*Prunus salicina*	GU968758.1
S1	*Prunus persica*	DQ269994.1
S31	*Prunus speciosa*	GU968645.1

Reviewer

ORIGINALITY REPORT

27%	21%	25%	3%
SIMILARITY INDEX	INTERNET SOURCES	PUBLICATIONS	STUDENT PAPERS

PRIMARY SOURCES

1	www.frontiersin.org<br>Internet Source	4%
2	www.tdx.cat<br>Internet Source	3%
3	Àngel Fernández i Martí, Toshio Hanada, José M. Alonso, Hisayo Yamane, Ryutaro Tao, Rafel Socias i Company. "The almond Sf haplotype shows a double expression despite its comprehensive genetic identity", Scientia Horticulturae, 2010<br>Publication	3%
4	hdl.handle.net<br>Internet Source	2%
5	link.springer.com<br>Internet Source	2%
6	Irita Kota-Dombrovska, Gunārs Lācis. "Evaluation of Self-Incompatibility Locus Diversity of Domestic Plum (Prunus Domestica L.) using DNA-Based S-Genotyping",	1%

Proceedings of the Latvian Academy of Sciences. Section B. Natural, Exact, and Applied Sciences, 2013
Publication

7	journal.ashspublications.org<br>Internet Source	1%
8	Kentaro Ono, Takashi Akagi, Takuya Morimoto, Ana Wünsch, Ryutaro Tao. "Genome Re-Sequencing of Diverse Sweet Cherry (Prunus avium) Individuals Reveals a Modifier Gene Mutation Conferring Pollen-Part Self-Compatibility", Plant and Cell Physiology, 2018<br>Publication	1%
9	Patrik Inderbitzin, Marilena Christopoulou, Dean Lavelle, Sebastian Reyes-Chin-Wo et al. "The LsVe1L allele provides a molecular marker for resistance to Verticillium dahliae race 1 in lettuce", BMC Plant Biology, 2019<br>Publication	1%
10	Shashi N. Goonetilleke, Adam E. Croxford, Timothy J. March, Michelle G. Wirthensohn, Maria Hrmova, Diane E. Mather. "Variation among S-locus haplotypes and among stylar RNases in almond", Scientific Reports, 2020<br>Publication	<1%
11	www.nature.com<br>Internet Source	<1%

12	www.mdpi.com<br>Internet Source	<1%
13	www.ishs.org<br>Internet Source	<1%
14	O. Kodad. "Identification and characterization of new *S*-alleles associated with self-incompatibility in almond", Plant Breeding, 12/2008<br>Publication	<1%
15	www.rosaceae.org<br>Internet Source	<1%
16	Angel Fernández i Martí. "A modifier locus affecting the expression of the S-RNase gene could be the cause of breakdown of self-incompatibility in almond", Sexual Plant Reproduction, 06/17/2009<br>Publication	<1%
17	bbs3.sekkaku.net<br>Internet Source	<1%
18	academic.oup.com<br>Internet Source	<1%
19	Fan, L.T.. "Comment on: An improved microkinetic model for the water-gas shift reaction on copper [Surf. Sci. 541 (2003) 21-30]", Surface Science, 20070601<br>Publication	<1%

20	Mo Zhu, Xiaoming Zhang, Kaichun Zhang, Lijie Jiang, Limin Zhang. "Development of a simple molecular marker specific for detecting the self-compatibleS4′ haplotype in sweet cherry (Prunus avium L.)", Plant Molecular Biology Reporter, 2012<br>Publication	<1%
21	citarea.cita-aragon.es<br>Internet Source	<1%
22	fjfsdata01prod.blob.core.windows.net<br>Internet Source	<1%
23	Cachi, A. M., and A. Wunsch. "Characterization and mapping of non-S gametophytic self-compatibility in sweet cherry (Prunus avium L.)", Journal of Experimental Botany, 2011.<br>Publication	<1%
24	Submitted to University of the Western Cape<br>Student Paper	<1%
25	M. E. Guerra. "*S-RNase* genotyping and incompatibility group assignment by PCR and pollination experiments in Japanese plum", Plant Breeding, 06/2009<br>Publication	<1%
26	Ossama Kodad, José M. Alonso, Angel Fernández i Martí, M. Margarida Oliveira, Rafel Socias i Company. "Molecular and physiological	<1%

identification of new S-alleles associated with self-(in)compatibility in local Spanish almond cultivars", Scientia Horticulturae, 2010
Publication

27	I. Kota, G. Lācis. "EVALUATION OF GENETIC DIVERSITY IN PLUM GERMPLASM COLLECTION USING DNA-BASED S-GENOTYPING", Acta Horticulturae, 2013<br>Publication	<1%
28	Juan A. Roldán, Rodrigo Quiroga, Ariel Goldraij. "Molecular and genetic characterization of novel S-RNases from a natural population of Nicotiana alata", Plant Cell Reports, 2010<br>Publication	<1%
29	elifesciences.org<br>Internet Source	<1%
30	"Fruit Breeding", Springer Science and Business Media LLC, 2012<br>Publication	<1%
31	"Fruits and Nuts", Springer Science and Business Media LLC, 2007<br>Publication	<1%
32	M.E. Guerra, A. Wünsch, J. Rodrigo, M. López-Corrales. "INFLUENCE OF POLLINATION ON THE LOW FRUIT SET IN JAPANESE PLUM", Acta Horticulturae, 2010<br>Publication	<1%

33	mafiadoc.com<br>Internet Source	<1%
34	Chao Gu, Xiao-Juan Zong, Jia-Wei Wang, Hai-Rong Wei, Qing-Zhong Liu. " Characterising novel and alleles in ", The Journal of Horticultural Science and Biotechnology, 2015<br>Publication	<1%
35	Zehra Tuğba Murathan, Salih Kafkas, Bayram Murat Asma, Hayat Topçu. " _allele identification and genetic diversity analysis of apricot cultivars ", The Journal of Horticultural Science and Biotechnology, 2017<br>Publication	<1%
36	www.haute-normandie.environnement.gouv.fr<br>Internet Source	<1%
37	Gu, Chao, Schuyler S Korban, and Y. Han. "Identification and characterization of S-RNase genes and S-genotypes in Prunus and Malus species", Canadian Journal of Plant Science, 2014.<br>Publication	<1%
38	Rafel Socias i Company, Ossama Kodad, Angel Fernández i Martí, José M. Alonso. "Mutations conferring self-compatibility in Prunus species: From deletions and insertions to epigenetic alterations", Scientia Horticulturae, 2015<br>Publication	<1%

39	Submitted to University of Leeds<br>Student Paper	<1%
40	aem.asm.org<br>Internet Source	<1%
41	worldwidescience.org<br>Internet Source	<1%
42	A. Hegedűs. "Sexual incompatibility in Rosaceae fruit tree species: molecular interactions and evolutionary dynamics", Biologia Plantarum, 01/07/2012<br>Publication	<1%
43	Gunars Lacis. "Identification of self-incompatibility (S) alleles in Latvian and Swedish sweet cherry genetic resources collections by PCR based typing", Euphytica, 03/2008<br>Publication	<1%
44	Li, Wei, Qing Yang, Zhaoyu Gu, Chuanbao Wu, Dong Meng, Jie Yu, Qiuju Chen, Yang Li, Hui Yuan, Dongmei Wang, and Tianzhong Li. "Molecular and genetic characterization of a self-compatible apple cultivar, �CAU-1�", Plant Science, 2016.<br>Publication	<1%
45	S. P. Vaughan. "Characterisation of novel S-alleles from cherry (Prunus avium L.)", Tree	<1%

Genetics & Genomes, 07/2008
Publication

46	bmcplantbiol.biomedcentral.com<br>Internet Source	<1%
47	docplayer.net<br>Internet Source	<1%
48	techagro.org<br>Internet Source	<1%
49	Àngel Fernández i Martí, Werner Howad, Ryutaro Tao, José Manuel Alonso Segura, Pere Arús, Rafel Socias i Company. "Identification of quantitative trait loci associated with self-compatibility in a Prunus species", Tree Genetics & Genomes, 2011<br>Publication	<1%
50	"Wild Crop Relatives: Genomic and Breeding Resources", Springer Science and Business Media LLC, 2011<br>Publication	<1%
51	Advances in Cellular and Molecular Biology of Plants, 1994.<br>Publication	<1%
52	Kodad, O.. "Influence of genotype, year and type of fruiting branches on the productive behaviour of almond", Scientia Horticulturae, 20060721	<1%

Publication

53 Laura Carrera. "Genomic characterization of self-incompatibility ribonucleases (S-RNases) in loquat (Eriobotrya japonica Lindl.) (Rosaceae, Pyrinae)", Molecular Breeding, 05/2009 <1%
Publication

54 Ossama Kodad, Rafel Socias i Company, José M. Alonso. "Unilateral recognition of the Sf allele in almond", Scientia Horticulturae, 2015 <1%
Publication

55 Xiao-yan ZHANG, Xue-sen CHEN, Qing-wei MENG, Yuan-mao JIANG, Yang ZHENG, Xiao-liu CHEN. "Transcript and Sequence Analysis of S-RNases in Self-Compatible Tetraploid Chinese Cherry (Prunus pseudocerasus L.)", Agricultural Sciences in China, 2010 <1%
Publication

56 A. Wünsch, R. Tao, J. I. Hormaza. "Self-compatibility in 'Cristobalina' sweet cherry is not associated with duplications or modified transcription levels of S-locus genes", Plant Cell Reports, 2010 <1%
Publication

57 Brittany L. Sutherland, Brandie M. Quarles, Laura F. Galloway. "Intercontinental dispersal and whole-genome duplication contribute to loss of self-incompatibility in a polyploid complex", <1%

American Journal of Botany, 2018
Publication

58	Ivana Glišić, Dragan Milatović, Radosav Cerović, Sanja Radičević, Milena Đorđević, Nebojša Milošević. "Examination of self-compatibility in promising plum (Prunus domestica L.) genotypes developed at the Fruit Research Institute, Čačak", Scientia Horticulturae, 2017<br>Publication	<1%
59	Laura Carrera, Javier Sanzol, Esteve Soler, María Herrero, Jose I. Hormaza. "Molecular S-genotyping and determination of S-RNase-based incompatibility groups in loquat [Eriobotrya japonica (Thunb.) Lindl.]", Euphytica, 2011<br>Publication	<1%
60	Nada Šurbanovski. "Self-incompatibility of Prunus tenella and evidence that reproductively isolated species of Prunus have different SFB alleles coupled with an identical S-RNase allele", The Plant Journal, 5/2007<br>Publication	<1%
61	Ortega E., Egea J., Cánovas J., Dicenta F.. "Pollen tube dynamics following half- and fully-compatible pollinations in self-compatible almond cultivars", Sexual Plant Reproduction,	<1%

2002
Publication

62 S. P. Vaughan, K. Russell, D. J. Sargent, K. R. Tobutt. "Isolation of S-locus F-box alleles in Prunus avium and their application in a novel method to determine self-incompatibility genotype", Theoretical and Applied Genetics, 2005 <1%
Publication

63 arizona.openrepository.com <1%
Internet Source

64 www.jstage.jst.go.jp <1%
Internet Source

65 www.pubmedcentral.nih.gov <1%
Internet Source

66 yorkspace.library.yorku.ca <1%
Internet Source

67 "Genetics and Genomics of Rosaceae", Springer Science and Business Media LLC, 2009 <1%
Publication

68 Angel Fernández i Martí, Thomas M. Gradziel, Rafel Socias i Company. "Methylation of the S f locus in almond is associated with S-RNase loss of function", Plant Molecular Biology, 2014 <1%
Publication

69	Company, Rafel Socias i, Ossama Kodad, Angel Fernández i Martí, and José M. Alonso. "Mutations conferring self-compatibility in Prunus species: From deletions and insertions to epigenetic alterations", Scientia Horticulturae, 2015.<br>Publication	<1%
70	Fernanda R. C. dos Santos, Maria I. Zucchi, Jong-Won Park, Thiago R. Benatti et al. "New Sugarcane Microsatellites and Target Region Amplification Polymorphism Primers Designed from Candidate Genes Related to Disease Resistance", Sugar Tech, 2016<br>Publication	<1%
71	Tineke Sonneveld, Kenneth R. Tobutt, Simon P. Vaughan, Timothy P. Robbins. " Loss of Pollen-Function in Two Self-Compatible Selections of Is Associated with Deletion/Mutation of an Haplotype–Specific F-Box Gene ", The Plant Cell, 2005<br>Publication	<1%
72	"Production Technology of Stone Fruits", Springer Science and Business Media LLC, 2021<br>Publication	<1%
73	Angel Fernandez i Marti, Christopher A. Saski, George A. Manganaris, Ksenija Gasic, Carlos H.	<1%

Crisosto. "Genomic Sequencing of Japanese Plum (Prunus salicina Lindl.) Mutants Provides a New Model for Rosaceae Fruit Ripening Studies", Frontiers in Plant Science, 2018

Publication

74	G. Chen, B. Zhang, Z. Zhao, Z. Sui, H. Zhang, Y. Xue. "'A life or death decision' for pollen tubes in S-RNase-based self-incompatibility", Journal of Experimental Botany, 2009<br>Publication	<1%
75	Halász, Júlia, Ossama Kodad, and Attila Hegedűs. "Identification of a recently active Prunus -specific non-autonomous Mutator element with considerable genome shaping force", The Plant Journal, 2014.<br>Publication	<1%
76	K. R. Tobutt. "A new self-compatibility haplotype in the sweet cherry 'Kronio', S5', attributable to a pollen-part mutation in the SFB gene", Journal of Experimental Botany, 11/26/2007<br>Publication	<1%
77	McClure, B., F. Cruz-Garcia, and C. Romero. "Compatibility and incompatibility in S-RNase-based systems", Annals of Botany, 2011.<br>Publication	<1%
78	S. Lekhchine, T. Bahi, Y. Soufi. "Field oriented control of a six phases induction motor", 2013	<1%

Eighth International Conference and Exhibition on Ecological Vehicles and Renewable Energies (EVER), 2013
Publication

79	Sexual Reproduction in Animals and Plants, 2014.<br>Publication	<1%

Exclude quotes	Off	Exclude matches	Off
Exclude bibliography	Off		
