# Supplementary material for: Evaluation of the S-locus in Prunus domestica, characterization, phylogeny and 3D modelling
Source: PLoS One. 2021 May 13;16(5):e0251305. doi: 10.1371/journal.pone.0251305 (PMC8118244; doi:10.1371/journal.pone.0251305)
Supplement: Attachment — Submitted filename: Response to Reviewers.docx [file pone.0251305.s004.docx]

Reviewer #1: In the current study, the authors have characterized the S-locus in Prunus domestica. Further, its phylogeny and 3D modelling have also been carried out. Overall, I do not see any major issue with the experimentation. However, only a few points can be addressed before further consideration.

λ Line 47-52, please support your statements with vital reference(s).

Done. We have added two new references.

λ Line 771/Table 1, remove “S” from the end of the caption, and define SC/SI in the table caption or as a footnote.

Done. We have added the definition of SC/SI

λ Currently, I could not find any information for MW407935- MW407936. Please follow the journal policy regarding data availability.

Done. We have request to NCBI to make our sequences publicly available and now are open to everyone.

λ Line 307, could you please provide us with microscopic images (within text or suppl. figure) of the pollen tube behavior in pistils.

Included a new picture (Picture 1) and labelled the rest following the new order.

λ Figure 3 quality is very poor (96 dpi), and the text is not clearly visible. Please provide an image with a higher resolution.

Done. Picture improved at 300dpi and tiff format

λ It would be nice to add the Ramachandran plot in figure 4 together with 3D models.

Done. We have added a new Ramachandran plot in figure 5(c).

λ During the screening, I found that several sentences are very similar to previous publications. The authors must rephrase the plagiarized text. Please see the attached report.

Done, we have removed and edited new sentences.

Reviewer #2: In this study, one of the important aspects of self-incompatible plant breeding has been investigated. Although the results are small, but enough innovation is provided.

Some comments and suggestions:
- Add references to the first paragraph of introduction.

Done. We have added two new references.

- Introduction is so long, so I suggest to remove/merge some sentences.

Done. We have e followed the reviewer advice and have shortened the introduction

- Discussion needs to improve.

The reviewer requested improvement to the Discussion without any further details. We have taken a close look and made a few changes including some reorganization that we feel makes the manuscript stronger and clearer.
